# Risk factors for pulmonary tuberculosis in Croatia: a matched case–control study

**DOI:** 10.1186/1471-2458-13-991

**Published:** 2013-10-21

**Authors:** Anamarija Jurcev-Savicevic, Rosanda Mulic, Bozica Ban, Karlo Kozul, Ljiljana Bacun-Ivcek, Jasna Valic, Gordana Popijac-Cesar, Snjezana Marinovic-Dunatov, Majda Gotovac, Aleksandar Simunovic

**Affiliations:** 1 Teaching Public Health Institute of Split and Dalmatia County, Vukovarska 46, 21 000 Split, Croatia; 2 School of Medicine, University of Split, Soltanska 2, 21000 Split, Croatia; 3 Public Health Institute “Dr Andrija Štampar”, Mirogojska cesta 16, 10000 Zagreb, Croatia; 4 Public Health Institute of Osječko-Baranjska County, F. Krežme 1, 31000 Osijek, Croatia; 5 Public Health Institute of Istarska County, Nazorova 23, 52100 Pula, Croatia; 6 Public Health Institute of Krapinsko-Zagorska County, Ivana Gorana Kovačića 1, 49250 Zlatar, Croatia; 7 Public Health Institute of Zadarska County, Kolovare 2, 23000 Zadar, Croatia; 8 Croatian National Institute of Public Health, Rockefellerova 7, 10000 Zagreb, Croatia

**Keywords:** Tuberculosis, Risk factors, Poverty, Diabetes, Malignant disease, Prevention, Intervention, Croatia

## Abstract

**Background:**

*Mycobacterium tuberculosis* is a necessary, but not sufficient, cause of tuberculosis. A number of studies have addressed the issue of risk factors for tuberculosis development. Croatia is a European country with an incidence rate of 14/100 000 which is slowly decreasing. The aim of this study is to evaluate the potential demographic, socioeconomic, behavioural and biological risk factors for tuberculosis in Croatia in comparison to other high-income, low-incidence European countries.

**Methods:**

A total of 300 tuberculosis patients were matched for age, sex and county of residence to 300 controls randomly selected from general practitioners’ registers. They were interviewed and their medical records were evaluated for variables broadly described as potential risk factors.

**Results:**

In multiple logistic regression, the following factors were significant: parents born in a particular neighbouring county (Bosnia and Herzegovina) (OR = 3.90, 95% CI 2.01-7.58), the lowest level of education (OR = 3.44, 95% CI 1.39-8.50), poor household equipment (OR = 4.72, 95% CI 1.51-14.76), unemployment (OR = 2.69, 95% CI 1.18-6.16), contact with tuberculosis (OR = 2.19, 95% CI 1.27-3.77), former (OR = 2.27, 95% CI 1.19-4.33) and current smoking habits (OR = 2.35, 95% CI 1.27-4.36), diabetes (OR = 2.38, 95% CI 1.05-5.38), a malignant disease (OR = 5.79, 95% CI 1.49-22.42), being underweight in the previous year (OR = 13.57, 95% CI 1.21-152.38).

**Conclusion:**

In our study, the identified risk groups for tuberculosis reflect a complex interaction between socioeconomic conditions, lifestyle and non-communicable diseases. Interventions focused on poverty will undoubtedly be useful, but not sufficient. Tuberculosis control would benefit from a combination of broad public health activities aimed at the prevention and control of risky lifestyles and non-communicable diseases, interventions outside the health sector, and efforts to constantly improve the Croatian national tuberculosis programme.

## Background

*Mycobacterium tuberculosis* is a necessary, but not sufficient, cause of tuberculosis. The risk of developing tuberculosis from an infected person is endogenous, depending on the integrity of cellular immune response and natural susceptibility. Various risk factors for tuberculosis (TB) development have been identified. It has been suggested that in European developed countries, as well as in the USA, the most important risk factors are immigration, homelessness, unemployment, a risky lifestyle such as illicit drug use, and the spread of the human immunodeficiency virus (HIV) infection [1-3]. In Eastern Europe after the break-up of the Soviet Union, political changes caused difficulties in the healthcare system and a lack of TB control which led to increased incidence, with low socioeconomic status and alcohol consumption as important risk factors [4-6]. In developing countries with a high TB burden, a TB epidemic is fuelled by poverty and rising HIV infection [7-10].

Croatia is a high-income country with a population of 4.4 million which has recently become a European Union Member State. TB incidence rates have been slowly decreasing, with a rate of 14/100 000 in 2011 [11-13]. However, an incidence rate <10/100 000, which was proclaimed in 1998 as a national goal, has not yet been achieved [14]. At the time of this study, the incidence rate was on average 23/100 000. One study exploring the epidemiology of tuberculosis in Croatia in the period from 1996 to 2005 revealed that the age patterns of TB cases corresponded to those of developed countries, with the highest incidence being at the oldest age, resulting probably from a reactivation of an old infection. A low proportion of drug-resistant and multidrug resistant tuberculosis (MDR-TB) was notified during the period observed (3.3% and 0.7%, respectively). Over a period of 20 years, TB was reported, on average, in 1.1% of AIDS cases annually. Croatia had a low-level HIV epidemic with 8.4% HIV-infected patients per 1 million. The low frequency of extrapulmonary tuberculosis (around 10%) might be attributed to the low frequency of HIV/AIDS cases in Croatia [13].

The previously described characteristics related to the TB epidemiological situation are generally still present nowadays.

Other high income, low-incidence European countries usually have a TB incidence rate less than 10/100 000, with the TB burden marked with HIV-coinfection, TB among immigrants, prisoners, illicit drug use, as well as with extrapulmonary TB localisation and MDR-TB cases [15-18].

Some routine epidemiological data have been collected in the long-standing and reliable Croatian notification system [12,13]. However, there is a need for deeper insight into the TB burden. The aim of this study is to determine the risk factors for pulmonary TB in order to properly focus healthcare financial funds related to TB control.

## Methods

We undertook a case–control study in seven randomly selected Croatian counties from 2006 to 2008. The idea was to include as many counties as needed to cover more than 50% of the Croatian population and more than 50% of the TB patients registered in the preceding year. This study started in eight randomly selected counties out of the total of 21, covering 60.8% of the Croatian population and 53.6% of all registered TB patients in the preceding year. In the course of the study, one county was later excluded along with all interviewees because the investigator was moved to another non-participating county. Therefore, seven counties with 48% of TB patients and 53.9% of the Croatian population were ultimately covered.

The study population was finally gathered on the basis of 300 cases and 300 controls.

We defined cases as adults (aged 15 years and older) with culture positive pulmonary TB. They were interviewed immediately after diagnosis. Cases that subsequently did not have a culture confirmed diagnosis were excluded from the study. The TB cases were consecutively enrolled until the sample size was achieved.

In Croatia, infectious TB patients have to be interviewed by epidemiologists, and were thus interviewed independently of this study, for contact tracing purposes. Consequently, these interviews were different and longer than those normally conducted. They were usually held in hospitals (Croatian patients generally have to be hospitalized during the initial phase or at least 2–3 weeks after the initiation of treatment) or in their homes (if they started TB treatment at home).

All the authors of this study are epidemiologists and have been involved in TB control at county level (including the interviewing of TB patients) and one author has been working at the national TB registry. The dual TB notification system (physician and laboratory notification) has been successfully implemented since 1998. This means that the epidemiologists (the authors of this study) would receive two notifications for a single laboratory-confirmed TB patient, one from the physician and another from the laboratory, the microscopy results immediately (24 hours) and the culture results upon cultivation. This notification system ensures that all confirmed cases were included in this study.

When all the cases were interviewed, one general practitioner from the corresponding county was randomly selected for each TB case. Primary healthcare, including general medicine, is covered by mandatory insurance provided by the Croatian health insurance fund, and general practitioners are its contracted partners. Therefore, general practice is provided free of charge for all insured Croatian citizens (98.3% of the total Croatian population at the time of this study) and 95% of the total population was registered in general practitioners’ registers [19]. The diagnostic procedures and drugs recommended by general practitioners are also mainly free of charge. Some co-payment may be required (usually covered by supplementary, non-mandatory insurance), but tuberculosis prevention, diagnosis and treatment are completely free of charge [14].

We selected four controls individually matched to cases by age (year of birth ±2), sex, and county of residence that had no history of TB from the database of general practitioners, taking into consideration the low response rate in other studies [5]. Only one control was interviewed, the first control in line. If that control needed to be excluded, the next in line was invited. Considering that all included TB patients were registered in the GPs’ registers from which the control group was selected, we believe that the cases and the controls came from the same source population.

The study was designed so that tuberculosis development in the control group would lead to the exclusion of such a participant. After interviewing the control group, it was found that none had developed tuberculosis in a two-month period. All new TB patients during that period would have been reported to the authors of this study who would have checked their names in the database of controls.

Several exclusion criteria were defined for both study groups (unavailability, wrong address, refusal to participate, serious illness, and death). The study was approved by the Ethics Committee of the Teaching Institute of Public Health of Split and Dalmatia County. Informed consent was obtained from all the patients, including parental consent from patients under the age of 18.

### Data collection

The same structured and pre-coded questionnaire was administered to all the subjects. The risk factors were selected by consensus of the authors and following analysis of similar studies. Many variables that broadly described demographic, socioeconomic, behavioural and biological conditions were evaluated as potential risk factors, as shown in Tables 1, 2, and 3. Household equipment (vehicle, refrigerator, air-conditioning, washing machine, cooker, television, personal computer, telephone) in different combinations was defined as poor (≤ 2 items, moderate (3–5 items), and good (≥ 6 items). Childbirth in both groups and tuberculosis for the cases in this study were excluded as reasons for hospitalization. The body mass index (BMI) (kg/m^2^) in the previous year was defined as: underweight (<18.5), normal weight (18.5 - 24.99), and overweight (≥25).

**Table 1:** Frequencies and bivariate logistic regression of demographic and socioeconomic variables of tuberculosis patients (N = 300) and controls (N = 300)

**Variable**	**Controls N (%)**	**Cases N (%)**	**Bivariate logistic regression**
			**OR (95% CI)**
Marital status			
With partner	205 (68.3)	174 (58)	1
Single	95 (31.7)	126 (42)	1.79 (1.21-2.65)
Country of birth of parents			
Croatia	261 (87)	209 (69.7)	1
Bosnia and herzegovina	31 (10.3)	73 (24.3)	2.86 (1.79-4.59)
Other	8 (2.7)	18 (6.0)	2.82 (1.15-6.93)
Nationality			
Croatian	283 (94.3)	271 (90.3)	1
Other	17 (5.7)	29 (9.7)	2.33 (1.07-5.09)
Level of education			
Higher education	55 (18.3)	29 (9.7)	1
Secondary school	165 (55.0)	126 (42.0)	1.53 (0.88-2.67)
No schooling or only elementary school	80 (26.7)	145 (48.3)	5.55 (2.89-10.65)
Housing *			
Ownership	282 (90.4)	257 (86.5)	1
Homeless/ institution/ subtenant	18 (6.0)	40 (13.5)	2.69 (1.42-5.09)
Crowding*†			
Yes	139 (46.6)	94 (34.2)	1
No	159 (53.4)	181 (65.8)	1.72 (1.21-2.44)
Household equipment*			
Good	190 (63.3)	100 (33.4)	1
Moderate	103 (34.3)	155 (51.8)	4.16 (2.64-6.57)
Poor	7 (2.3)	44 (14.7)	18.82 (7.33-48.33)
Central water supply			
Yes	265 (88.3)	245 (81.7)	1
No	35 (11.7)	55 (18.3)	1.83 (1.11-3.01)
Central sewage system			
Yes	198 (66.0)	185 (61.7)	1
No	102 (34.0)	115 (38.3)	1.24 (0.87-1.76)
Employment			
Employed	136 (45.3)	104 (34.7)	1
Unemployed	22 (7.3)	70 (23.3)	3.82 (2.17-6.70)
Housewife/ retired/ student/ other	142 (47.3)	126 (42.0)	1.11 (0.66-1.86)
Total personal monthly income‡			
> Average salary	55 (18.5)	37 (12.6)	1
Minimal –average salary	137 (46.0)	104 (35.4)	1.05 (0.65-1.70)
≤ Minimal salary	106 (35.6)	153 (52.0)	2.07 (1.26-3.40)
Food shortage			
No	288 (96.0)	274 (91.3)	1
Yes	12 (4.0)	26 (8.7)	2.17 (1.09-4.29)

* Missing values.

† Defined as less than one inhabitable room per person.

‡ According to the Croatian Bureau of Statistics.

OR = Odds ratio, CI = Confidence interval.

**Table 2:** Frequencies and bivariate logistic regression of behavioural variables of tuberculosis patients (N = 300) and controls (N = 300)

**Variable**	**Controls N (%)**	**Cases N (%)**	**Bivariate logistic regression**
			**OR (95% CI)**
Being in prison/ pre-trial detention centre*			
No	286 (95.7)	280 (93.3)	1
Yes	13 (4.3)	20 (6.7)	1.58 (0.77-3.26)
Contact with tuberculosis			
No	212 (70.7)	170 (56.7)	1
Yes	88 (29.3)	130 (43.3)	1.91 (1.34-2.73)
Smoking status*			
Never	151 (50.3)	93 (31.1)	1
Ex-smoker	81 (27.0)	99 (33.1)	2.55 (1.60-4.05)
Current	68 (22.7)	107 (35.8)	3.14 (1.98-4.98)
Age of starting to smoke, years old			
≥ 25	10 (6.7)	6 (2.9)	1
20-24	38 (25.5)	26 (12.6)	0.78 (0.13-4.88)
≤19	101 (67.8)	174 (84.5)	3.17 (0.58-17.33)
Average number of cigarettes daily			
1-10	50 (34.0)	18 (8.8)	1
11-20	64 (43.5)	108 (52.7)	14.05 (3.31-59.71)
≥21	33 (22.4)	79 (38.5)	17.18 (3.80-77.61)
Total smoking time, years			
≤10	32 (21.5)	25 (12.1)	1
11-20	45 (30.2)	44 (21.4)	1.46 (0.53-4.07)
≥21	72 (48.3)	137 (66.5)	6.06 (1.81-20.27)
Passive smoking (for non-smokers)			
No	88 (58.3)	37 (39.8)	1
Yes	63 (41.7)	56 (60.2)	2.86 (1.21-6.76)
Alcohol consumption			
Non-consumer	145 (48.3)	125 (41.7)	1
Ex-consumer	25 (8.3)	35 (11.7)	1.71 (0.97-3.03)
Current consumer	130 (43.3)	140 (46.7)	1.38 (0.93-2.05)
Frequency of alcohol consumption, last 12 months			
Less than once a week	44 (28.4)	26 (14.9)	1
At least once a week	59 (38.1)	69 (39.4)	2.42 (0.97-6.07)
Daily	52 (33.5)	80 (45.7)	3.86 (1.56-9.55)
Being ever drunk			
No	169 (56.3)	162 (54.0)	1
Yes, rarely	112 (37.3)	107 (35.7)	1.03 (0.70-1.52)
Yes, often	19 (6.3)	31 (10.3)	1.78 (0.93-3.43)
Ever used drugs			
No	293 (97.7)	285 (95.0)	1
Yes	7 (2.3)	15 (5.0)	2.14 (0.87-5.25)
Illicit drug use			
No	299 (99.7)	297 (99.0)	1
Yes	1 (0.3)	3 (1.0)	3.00 (0.31-28.84)

*One missing value.

OR = Odds ratio, CI = Confidence interval.

**Table 3:** Frequencies and bivariate logistic regression of health related variables of tuberculosis patients (N = 300) and controls (N = 300)

**Variable**	**Controls N (%)**	**Cases N (%)**	**Bivariate logistic regression**
			**OR (95% CI)**
Previous hospitalizations			
No	112 (37.3)	110 (36.7)	1
Yes	188 (62.7)	190 (63.3)	1.03 (0.73-1.45)
Diabetes			
No	283 (94.3)	269 (89.7)	1
Yes	17 (5.7)	31 (10.3)	1,93 (1,04-3,60)
Malignant disease			
No	295 (98.3)	285 (95.0)	1
Yes	5 (1.7)	15 (5.0)	3.00 (1.09-8.25)
Chronic renal failure/ dialysis			
No	299 (99.7)	298 (99.3)	1
Yes	1 (0.3)	2 (0.7)	2.00 (0.18-22.06)
Transplantation			
No	299 (99.7)	299 (99.7)	1
Yes	1 (0.3)	1 (0.3)	1.00 (0.62-15.99)
HIV infection			
No	300 (100)	299 (99.7)	-
Yes	0	1 (0.3)	-
Immunosuppressive therapy*			
No	293 (97.7)	289 (96.3)	1
Yes	7 (2.3)	11 (3.7)	1.67 (0.60-4.58)
Other chronic diseases			
No	167 (55.7)	170 (56.7)	1
Yes	133 (44.3)	130 (43.3)	0.95 (0.65-1.38)
Co morbidity†			
Yes	141 (47.0)	144 (48.0)	1
No	159 (53.0)	156 (52.0)	1.05 (0.73-1.53)
Body mass index in the previous year ‡			
Overweight	188 (63.1)	112 (38.2)	1
Normal weight	108 (36.2)	171 (58.4)	2.83 (1.94-4.12)
Underweight	2 (0.7)	10 (3.4)	9.99 (2.09-47.85)

* Unrelated to the above-mentioned diseases.

† Any mentioned disease.

‡ missing values.

OR = Odds Ratio, CI = Confidence Interval.

A team of trained epidemiologists interviewed the cases in hospital or in their homes. The controls received a telephone / personal invitation to participate and a time was arranged for an interview. The interviewers made intensive efforts to reach the first controls, but if they could not be interviewed, the next one was invited.

### Statistical analysis

It was determined that 283 cases and an equal number of controls should be recruited to achieve 80% power to detect and an odds ratio of 2.0 at the level of significance of 5% if 10% of the general population were exposed to the risk factor [20].

The strength of the association of potential predictor variables for the occurrence of tuberculosis was analyzed by bivariate and multiple stepwise conditional logistic regressions. To include predictive variables in an initial multivariate model, statistical significance was determined at α = 0.20. The following steps in the multiple logistic regression retained predictors with a p value < 0.05, while other predictors were excluded until a model was built with all predictors significant at < 0.05. If the re-inclusion of an excluded predictor changed the odds ratios of the retained predictors by more than 20%, the excluded predictor was returned to the final multiple model to neutralize the effect of indirect connections. All variables were checked for co-linearity.

The statistical analysis was made using STATA/IC ver.11.1 (StataCorp. 2009. Stata Statistical Software: Release 11. College Station, TX: StataCorp LP).

## Results

Among 311 cases fulfilling the inclusion criteria, 11 were excluded (three for unavailability, three for death, three for serious illness, and two for refusal to participate).

Among the control group, 75 participants were excluded, 40 participants from the first potential controls, 23 from the second, 9 from the third, and 3 participants from the fourth potential controls, where the interviewer subsequently had to seek 3 controls as a fifth choice.

**Figure 1:**
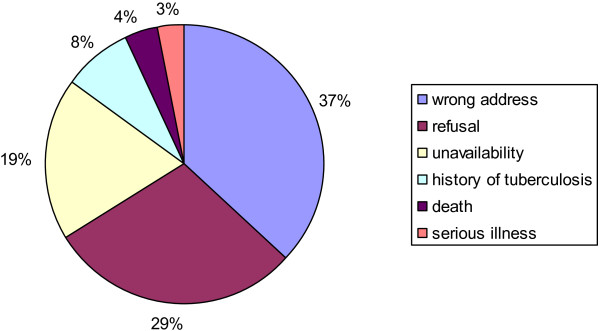
Reasons for exclusion of individuals from the control group (N = 75).

The most common reason for the exclusion of controls was the wrong address (37%). All reasons for exclusion are presented in Figure 1.

The frequencies of demographic, socioeconomic, behavioural and biological determinants, as well as the bivariate logistic regression results, are presented in Tables 1, 2, and 3.

Some of these variables remained significant in the multiple analysis, such as parents born in Bosnia and Herzegovina (OR 3.90, 95% CI 2.00-7.58), the lowest level of education (OR 3.44, 95% CI 1.39-8.50), moderate (OR 4.18, 95% CI 2.27-7.71) and poor (OR 4.73, 95% CI 1.51-14.76) household equipment, unemployment (OR 2.69, 95% CI 1.18-6.16), contact with tuberculosis (OR 2.19, 95% CI 1.27-3.77), former (OR 2.27, 95% CI 1.19-4.33) and current smoking (OR 2.35, 95% CI 1.27-4.36), diabetes (OR 2.38, 95% CI 1.05-5.38), a malignant disease (OR 5.79, 95% CI 1.49-22.42), normal weight (OR 2.88, 95% CI 1.74-4.76) and being underweight (OR 13.57, 95% CI 1.21-152.38) in the previous year (Table 4).

**Table 4:** Multiple logistic regression of significant variables of cases with pulmonary tuberculosis (N = 300) and controls (N = 300)

**Variable**	**Multiple logistic regression**
	**OR (95% CI)**
Country of birth of parents	
Croatia	1
Bosnia and Herzegovina	3.90 (2.00-7.58)
Other	2.13 (0.64-7.13)
Level of education	
Higher education	1
Secondary school	1.35 (0.64-2.85)
No schooling or only elementary school	3.44 (1.39-8.50)
Household equipment	
Good	1
Moderate	4.18 (2.27-7.71)
Poor	4.73 (1.51-14.76)
Employment	
Employed	1
Unemployed	2.69 (1.18-6.16)
Housewife/ retired/ student/ other	0.56 (0.26-1.22)
Smoking status	
Never	1
Ex-smoker	2.27 (1.19-4.33)
Current	2.35 (1.27-4.36)
Contact with tuberculosis	
No	1
Yes	2.19 (1.27-3.77)
Malignant disease	
No	1
Yes	5.79 (1.49-22.42)
Diabetes	
No	1
Yes	2.38 (1.05-5.38)
Body mass index in the previous year	
Overweight	1
Normal weight	2.88 (1.74-4.76)
Underweight	13.57 (1.21-152.38)

OR = Odds ratio, CI = Confidence interval.

## Discussion

This is the first study of risk factors for pulmonary TB in Croatia. The social determinants of tuberculosis in the study population are related to unemployment, the lowest level of education and poor household equipment, and to some extent malnutrition. Poverty undoubtedly contributes to the incidence of tuberculosis through increased progression from infection to disease due to poor diet or stress, and greater difficulties in using health services [7].

Former and current smoking, as well as contact with tuberculosis, was significant among the behavioural factors. This merits attention because smoking is a highly prevalent hazardous habit, both in our population and in the world, and is also socially accepted. According to recently published data from the Croatian national follow-up cohort study, CroHort, 21% of respondents were smokers in 2008 [21].

Passive exposure to tobacco smoke in non-smokers in a bivariate analysis was also associated with tuberculosis. Those exposed to passive smoke inhaled similar toxic substances as active smokers, although in different concentrations. Passive smoking has a smaller effect on the morbidity of tuberculosis at the individual level, but it can have a much greater impact at the population level, because anyone who breathes the same air can be exposed, whether a smoker or non-smoker [22]. This relationship is important because smoking is one of the habits on which influence may be exerted. In Croatia, this association may be emphasized as an integral part of the prevention of cancer and cardiovascular diseases that are more prevalent than tuberculosis. Anti-smoking campaigns, legislation, restrictions on smoking in public places and institutions, and high tobacco prices are likely to contribute to a decrease in the prevalence of smoking.

Various biological factors (chronic renal failure / dialysis, transplantation, HIV infection, immunosuppressive therapy) that were proven in other studies [23-26] were not confirmed here, probably due to the fact that these conditions are not highly prevalent in this population.

However, it was found that diabetes and malignant diseases were associated with the incidence of tuberculosis. All of them suppress the cellular immune function, a key defence mechanism against *M. tuberculosis*[27-29].

The association of diabetes and TB has been observed in several studies, regardless of the design and the geographic area in which the studies were conducted, and the incidence of tuberculosis. A recent systematic review showed that diabetes carried a relative risk of 3.11 in the cohort studies, while in the case–control studies the odds ratios were heterogeneous, ranging from 1.16 to 7.83 [27]. This study shows that the likelihood of developing tuberculosis in patients with diabetes is 2.4 times higher than in the general population. Therefore, the national tuberculosis programme can benefit from the active search for and treatment of latent tuberculosis infection (LTBI) in diabetics, and from the appropriate diagnosis and treatment of diabetes [30].

The significant occurrence of TB in patients with malignant diseases is explained by a weakened immune system due to the primary disease and the influence of anticancer therapy [28,31]. Similar symptoms and overlapping clinical features, association with previous fibro calcified lesions in the lungs, and unclear LTBI testing results make the diagnosis of TB and LTBI in cancer patients demanding. On the other hand, the increased risk of TB in patients with a malignant disease makes these patients a target group for LTBI treatment, particularly because the number of new cases of malignant disease is increasing in Croatia [30,32]. High risk was observed in a low BMI, which was also found elsewhere [29,33]. It was found that the incidence of TB decreased with an increase in the BMI. This trend was almost linear on a logarithmic scale, regardless of gender and age [7,34]. It was calculated in one study that the relative risk of undernourished people developing TB was 6–10 [29]. This study also shows that a low and, to a lesser extent, a normal BMI, carried a higher risk than a high BMI. Although the number of undernourished subjects in both study groups was not high, the frequency of overweight participants was almost three times higher among those in the control group than in the cases. Malnutrition is an important cause of acquired immune dysfunction that can be managed by appropriate interventions [29].

Unlike most other high income, low-incidence European countries, HIV-coinfection and TB among drug addicts and prisoners do not seem to play an important role in TB epidemiology in Croatia [11,16,17].

These results might be more significant today in terms of unemployment, diabetes and malignant diseases since their burden in Croatia is increasing, while smoking, which is decreasing, might be less relevant as a TB risk factor [21,32,35].

The results of this study should be interpreted in the light of some limitations. Data on height and weight were taken from the participants’ recall, and it is possible that the BMI data were not entirely accurate. The studies that have explored uncommon risk factors had a larger sample size, mostly based on data from a TB register, or a dialysis or transplantation register and the like. Therefore, it is possible that the sample size, when it comes to factors that are present in a population of less than 10%, is insufficient to detect association.

Unlike some studies, the control group sample was selected from the general population rather than from other hospital patients, friends or relatives of TB patients, thereby enabling the analysis of more potential risk factors. Control sampling allows us to believe that the control group represents the total Croatian population. Criteria for the inclusion of patients were strict and allowed only culture-confirmed pulmonary cases. The survey was conducted by skilled physicians who perform surveys and field work on a daily basis.

## Conclusion

The public health dimension of TB in Croatia is complex, with some demographic, socioeconomic, behavioural and biological factors being important. Interventions focused on poverty are undoubtedly useful, but not sufficient. TB control should benefit from interventions against risky lifestyles and the prevention of chronic non-communicable diseases. The national tuberculosis programme within its traditional roles and responsibilities does not incorporate such actions. Therefore, the fight against TB should be accompanied by the additional participation of any structures that tailor health policy and by the involvement of the entire community. Rapid and appropriate diagnosis and the treatment of risk groups may reduce their importance in modern TB epidemiology. A combination of broad public health activities aimed at the prevention and control of non-communicable diseases, interventions outside the health sector and efforts to constantly improve the National Tuberculosis Programme can bring us closer to eliminating TB.

## Competing interests

The authors declare that they have no competing interests.

## Authors’ contributions

AJS produced the concept and design, participated in the collection, analysis and interpretation of data, and drafted the manuscript. RM contributed to the concept and design, analysis and interpretation of data, and critically revised the manuscript for important intellectual content. BB, KK, LJBI, JV, GPC, SMD, MG participated in the data collection, and revised critically the manuscript for important intellectual content. AS participated in the analysis and interpretation of data and critically revised the manuscript for important intellectual content. All authors read and approved the final manuscript.

## Authors’ information

All authors have been deeply involved in TB control in Croatia from different standpoints. AJS, BB, KK, LJBI, JV, GPC and SMD are regional TB managers. MG is young epidemiologist, AS is a national TB manager and RM is an experienced epidemiologist with a strong scientific background. AJS is continuously conducting and publishing studies on different aspects of TB control.

## Pre-publication history

The pre-publication history for this paper can be accessed here:

http://www.biomedcentral.com/1471-2458/13/991/prepub
